# Agreement between equation‐derived body fat estimator and bioelectrical impedance analysis for body fat measurement in middle‐aged southern Indians

**DOI:** 10.14814/phy2.70095

**Published:** 2024-10-21

**Authors:** Chiranjeevi Kumar Endukuru, Girwar Singh Gaur, Dhanalakshmi Yerrabelli, Jayaprakash Sahoo, Balasubramaniyan Vairappan

**Affiliations:** ^1^ Department of Physiology, School of Medicine and Dentistry University of Central Lancashire Preston Lancashire UK; ^2^ Department of Physiology Jawaharlal Institute of Postgraduate Medical Education and Research (JIPMER) Puducherry India; ^3^ Department of Endocrinology Jawaharlal Institute of Postgraduate Medical Education and Research (JIPMER) Puducherry India; ^4^ Department of Biochemistry Jawaharlal Institute of Postgraduate Medical Education and Research (JIPMER) Puducherry India

**Keywords:** bioelectrical impedance analysis, body composition, body fat percentage, cardiometabolic risk, CUN‐BAE, metabolic syndrome

## Abstract

Excess body fat (BF) contributes to metabolic syndrome (MetS). The Clínica Universidad de Navarra—Body Adiposity Estimator (CUN‐BAE) is an equation‐derived body fat estimator proposed to assess BF. However, its efficiency compared to the standard method is unknown. We aimed to compare the efficacy of CUN‐BAE with the standard method in estimating BF in southern Indians. We included 351 subjects, with 166 MetS patients and 185 non‐MetS subjects. BF was obtained from the standard bioelectrical impedance analysis (BIA) method and measured by CUN‐BAE in the same subjects. We compared the efficacy of CUN‐BAE in estimating BF with that of BIA via Bland–Altman plots, intraclass correlation coefficients, concordance correlation coefficients and the kappa index. The mean body fat percentage (BF%) values measured by BIA and CUN‐BAE in all the subjects were 28.91 ± 8.94 and 29.22 ± 8.63, respectively. We observed significant absolute agreement between CUN‐BAE and BIA for BF%. BIA and CUN‐BAE showed good reproducibility for BF%. CUN‐BAE had accuracy comparable to BIA for detecting MetS using BF%. Our findings indicate that CUN‐BAE provides precise BF estimates similar to the BIA method, making it suitable for routine clinical practice when access to BF measurement devices is limited.

## 1 INTRODUCTION

Metabolic syndrome (MetS) is increasingly common worldwide, and the risk of developing MetS rises significantly with a higher body mass index (BMI) (Grundy, 2008). MetS is a cluster of risk factors that increase the likelihood of developing type 2 diabetes mellitus (T2DM) and cardiovascular diseases (CVDs). It encompasses crucial factors such as abdominal obesity, dyslipidemia, hyperglycemia, and hypertension. Typically, individuals with MetS are overweight or obese, distinguished by an excess of body fat (BF), which is linked to chronic conditions such as T2DM and CVD (Eckel et al., 2010). Research suggests that evaluating the body fat percentage (BF%) provides a nuanced indication of overall health. Asian Indians have more subcutaneous and intra‐abdominal fat than individuals of European descent despite having lower body weight, BMI, fat‐free mass (FFM), and shorter stature. (Misra & Khurana, 2009). Excess adiposity induces metabolic and endocrine changes, leading to cardiometabolic disturbances and increased morbidity and mortality (Misra et al., 2010). The preservation of FFM has demonstrated its importance in addressing these cardiometabolic issues. Consequently, further attention should be given to exploring the relationships between BF% and associated metabolic risk factors.

Although there is no direct way to measure BF, various indirect techniques are used to estimate BF%. These techniques include skin‐fold thickness measurements, magnetic resonance imaging (MRI), air displacement plethysmography (ADP), dual‐energy x‐ray absorptiometry (DXA), and bioelectrical impedance analysis (BIA). Although these techniques have been verified against established methods for body composition and are commonly used to assess BF% (Das & Sai Krupa, 2005), their application in clinical practice is hindered by cost, safety, and complexity, regardless of their accuracy. Among these methods, skinfold thickness measurements are more widely used to estimate BF cost‐effectively, especially in epidemiologic studies. Although skinfold calipers are very affordable and measurements can be taken quickly, the method requires practice and basic knowledge of anatomy. Additionally, some people do not enjoy having their fat pinched. Therefore, there is a need for more straightforward methods to assess BF% that are more accurate, reliable, less expensive, and less invasive.

Owing to its unique relationship with total BF content and metabolic risk factors, BMI, waist circumference (WC), and other traditional anthropometric measures are helpful and sufficient for assessing adiposity at a population level. However, their accuracy and utility can diminish when applied to individuals due to variations in body composition and fat distribution. BMI is the most popular tool for assessing BF. However, BMI has many proven flaws, including failure to reflect age, gender, and ethnicity‐related variations in BF and FFM. Furthermore, individuals with substantial muscle mass tend to be misclassified as overweight or obese (Camhi et al., 2011; Gómez‐Ambrosi, Silva, Galofré, et al., 2012). BMI is often used to describe obesity in extensive studies. However, it may not accurately predict an individual's body fatness. In more recent studies, WC has been used to address the limitations of BMI. However, WC has limitations, such as not accounting for a person's weight and height, which can misinterpret obesity status in short or tall individuals (Bergman et al., 2011; Camhi et al., 2011). Skinfold thickness measures are used to compensate for the shortcomings of WC and BMI. It measures BF in all ages, including neonates, and is a simple, inexpensive, and non‐invasive method (Peterson et al., 2003). However, little information has been found on its validity. Thus, choosing the correct measure depends on the specific goal, whether for monitoring population health or assessing individuals clinically is essential. This establishes an urgent need to prioritize safe approaches involving equations to assess BF% reliably. This approach will help adequately evaluate and manage healthcare. Henceforth, novel adiposity measures such as the Clínica Universidad de Navarra—Body Adiposity Estimator (CUN‐BAE) and body adiposity index (BAI) have been suggested for enhanced precision in estimating BF% (Bergman et al., 2011; Gómez‐Ambrosi, Silva, Catalán, et al., 2012). The BAI focuses on the hip circumference (HC) and height, which correlate well with BF%. When men and women are evaluated individually, the BAI may underestimate or overestimate BF% owing to the stronger correlation between HC and BF% in women (Shin et al., 2017).

The CUN‐BAE is a novel predictive equation formulated by Gómez‐Ambrosi, Silva, Catalán, et al. (2012) to assess BF%. This equation considers age, gender, and BMI and deserves special attention because of its strong correlation with ADP‐measured BF% (Gómez‐Ambrosi, Silva, Catalán, et al., 2012). Moreover, Gomez et al. reported a stronger correlation of the CUN‐BAE equation with cardiometabolic risk factors compared to BMI and WC (Gómez‐Ambrosi, Silva, Catalán, et al., 2012). Lara et al. conducted a study on Caucasian subjects aged 61–84 years and reported that BF% measurements obtained via CUN‐BAE were similar to those obtained via ADP but not DXA (Lara et al., 2014). Among the various anthropometric variables, Vinknes et al. and Fuster‐Parra et al. reported that CUN‐BAE had the most significant association with BF%, as measured by BIA (*r* = 0.86) and DXA (*r* = 0.88) in Caucasians and Hordaland men and women aged 18–65 years (Fuster‐Parra et al., 2015; Vinknes et al., 2017). A southern Indian study used a QuadScan 4000 Bodystat analyzer (Isle of Man, United Kingdom) to assess body composition and reported significantly higher BF%, fat mass index (FMI) and lower lean body percentage in the MetS group than in the non‐MetS group (Endukuru et al., 2020). Numerous studies have shown the effectiveness of the QuadScan 4000 to assess and validate body composition (Endukuru et al., 2020; Fuster‐Parra et al., 2015; Gómez‐Ambrosi, Silva, Catalán, et al., 2012; Lara et al., 2014). However, studies that have validated the accuracy of CUN‐BAE with BIA via the QuadScan 4000 are scarce. Moreover, studies comparing the performance of CUN‐BAE with BIA via a QuadScan 4000 analyzer for measuring BF% in MetS and non‐MetS subjects in southern India remain elusive.

This study aimed to assess the effectiveness of CUN‐BAE in estimating BF% and to compare the agreement between the CUN‐BAE and BIA methods in individuals with MetS and without MetS.

## 2 MATERIALS AND METHODS

### 2.1 Study design and setting

We carried out this cross‐sectional study at the Puducherry Teaching Hospital in India. A total of 351 participants, aged 21–60, provided written informed consent before being included in the study, following the ethical principles outlined in the Declaration of Helsinki. Before consent, participants were fully informed about the study's nature, purpose, potential risks, and benefits. The Institutional Ethics Committee (Human Studies) approved the study. We recruited 166 MetS patients from the endocrine outpatient department who met the inclusion criteria after the initial screening. The National Cholesterol Education Program Adult Treatment Panel III (NCEP ATP III) (Eckel et al., 2010; Grundy, 2008) defines MetS as having three of the following five conditions such as higher WC, elevated serum triglycerides (TG), hyperglycemia, increased blood pressure ≥130/85 mmHg, and reduced HDL cholesterol. The non‐MetS group comprised 185 volunteers matched by age and gender.

### 2.2 Anthropometric, blood pressure, and biochemical profiles

We used a step‐by‐step World Health Organization (WHO) guide for accurate anthropometric measurements. We measured the subject's height and weight via a stadiometer and weighing scale. BMI is calculated via the Quetelet index, which divides an individual's weight in kilograms by their height in square meters. We used a non‐stretchable measuring tape to measure the WC around the narrowest point of the abdomen at the end of expiration and the HC around the buttocks at its widest point. The waist‐to‐hip ratio (WHR) and waist‐to‐height ratio (WHtR) were also computed. We recorded both arms' blood pressure (BP) three times via an Omron automated device after 5–10 min of sitting upright. We used commercially available kits and an auto‐analyzer to measure blood glucose and lipid profiles.

### 2.3 Assessment of body composition via QuadScan 4000 (BIA)

BIA is a tool that measures bioelectrical resistive impedance (R), which is used to assess body composition (Kyle et al., 2004). This approach is thought to be safe and accurate. BIA works on the theory that fat‐free tissue (primarily electrolyte‐containing water) conducts electricity better than fat (which acts as an insulator). The body's impedance is thus especially measured by low‐impedance lean tissues. A Bodystat® instrument (Model QuadScan 4000®, Isle of Man, United Kingdom) was used to achieve 5/50/100/200 kHz multiple frequency measurements. The QuadScan 4000 analysis tool is battery‐powered and straightforward, with no technical knowledge needed. The instrument has been precision‐engineered to the highest quality specifications using electronic technology, providing reliable and effective body composition measurements. The study participants fasted for 4 h and refrained from exercise for 24 h before the test. The test was conducted in a standard setting (quiet and ambient). The study participants were instructed to remain supine for 10 min with no body parts touching each other. The electrodes were attached to their hand and foot dorsal surfaces, close to the metacarpal–phalangeal and metatarsal‐phalangeal joints. Physical parameters such as height, weight, WC, HC, physical activity status, age, and gender were recorded to obtain body composition measurements.

### 2.4 Assessment of BF% by CUN‐BAE

Gómez et al. formulated the CUN‐BAE equation, which is used to measure BF% (Gómez‐Ambrosi, Silva, Catalán, et al., 2012): BF% = −44.988 + (0.503 × age) + (10.689 × gender) + (3.172 × BMI) − (0.026 × BMI^2^) + (0.181 × BMI × gender) − (0.02 × BMI × age) − (0.005 × BMI^2^ × gender) + (0.00021 × BMI^2^ × age).

This equation measures age in years, and gender is codified as men = 0 and women = 1.

### 2.5 Statistical analysis

We performed statistical analysis using the Statistical Package of Social Science (SPSS) for Windows version 20.00 (SPSS Inc., Chicago, IL, USA). Descriptive statistics were used to analyze the data, and we tested the normality of the data via the Kolmogorov–Smirnov test. We calculated the mean and standard deviation for normally distributed data. For non‐normally distributed data, we calculated the median and interquartile range. Comparisons were made using the two‐tailed Student's independent *t*‐test (parametric) and the Mann–Whitney *U*‐test (non‐parametric). Categorical (dichotomous) data were assessed via the chi‐square (*χ* ^2^) test. Analysis of covariance (ANCOVA) and linear regression were used to adjust BF% values by BIA for age, gender, and BMI. We used a paired *t*‐test to examine the mean differences between the two methods. We used Bland–Altman plot analysis (BA) to assess the degree of absolute agreement. In the BA plot, the *y*‐axis represents the difference between the two methods (CUN‐BAE—BIA), and the *x*‐axis represents the average of the two measurements ([CUN‐BAE + BIA]/2). We also determined the upper and lower limits of agreement by calculating the mean difference ± 1.96 SD.

We used the interclass correlation coefficient (ICC) to test the reproducibility of the BF% measured by CUN‐BAE compared with BIA. An ICC value below 0.4 indicates poor reproducibility, an ICC between 0.4 and 0.7 indicates moderate reproducibility and an ICC above 0.7 indicates good reproducibility. The Lin's concordance correlation coefficient (CCC) and the 95% confidence interval (95%CI) were calculated. The CCC is a measure that assesses precision and accuracy compared to the perfect concordance line. A CCC value of 1 indicates ideal concordance, while a CCC >0.900 indicates excellent concordance, 0.600–0.900 indicates moderate concordance, and <0.600 indicates poor concordance. We calculated the kappa coefficient and 95% confidence interval (95% CI) for both methods to classify participants as having MetS based on BF%. The kappa coefficient or *K* value can be interpreted as <20: poor; 21–40: fair; 41–60: moderate; 61–80: good; and 81–100: very good. We evaluated the relationship between the CUN‐BAE and BIA for BF% using Pearson's correlation coefficients. Additionally, we assessed the strength of the association of BF% estimated and measured by CUN‐BAE and BIA with MetS‐related parameters using Spearman's correlation coefficients. We also explored the performance of BIA and CUN‐BAE in identifying MetS by analyzing the area under the curve (AUC) of the receiver operating characteristic curve (ROC). All the analyses were conducted separately for each gender and grouped by category (MetS patients and non‐MetS subjects). We considered a *p*‐value of less than 0.05 to indicate statistical significance.

## 3 RESULTS

### 3.1 Demographic and anthropometric data

Table 1 provides the demographic, anthropometric and MetS‐related data of non‐MetS and MetS subjects stratified by gender. There were no significant differences between the non‐MetS and MetS groups in terms of age, smoking status, alcohol intake, or family history of metabolic diseases. However, female MetS patients had a greater prevalence of a family history of HTN (Table 1). There was a greater prevalence of smoking and alcohol consumption among men in both the non‐MetS and MetS groups. All MetS components were significantly more prevalent in both genders in the MetS group than in the non‐MetS group (Table 1). Male MetS subjects predominantly presented with hyperglycemia, whereas females presented increased abdominal obesity. Weight, BMI, WC, HC, WHR, and WHtR were significantly higher (*p* = 0.001) in the MetS group than in the non‐MetS group for both genders. Additionally, the participants in the MetS group had impaired glucose metabolism and dyslipidemia (Table 1). HDL‐C levels were lower, and prehypertensive status was observed in both genders of the MetS group. Compared with the non‐MetS group in both genders, the MetS group had substantially greater BF% and fat mass and lower lean body percentage, as estimated by BIA and CUN‐BAE (Table 2).

**TABLE 1:** Basal demographic, anthropometric, and MetS‐related parameters in non‐MetS and MetS subjects stratified by gender.

Variables	Male (*n* = 181)			Female (*n* = 170)		
	No MetS (*n* = 98)	MetS (*n* = 83)	*p*‐value	No MetS (*n* = 87)	MetS (*n* = 83)	*p*‐value
Age (years) ^ƥ^	44.0 (39.75–49.0)	47.0 (42.0–51.0)	0.119	49.0 (41.0–58.0)	48.0 (41.0–51.0)	0.140
Smoking: *n* (%) ^¶^	28 (28.6)	29 (34.9)	0.358	4 (4.6)	4 (4.8)	0.946
Alcohol intake: *n* (%) ^¶^	51 (52.0)	43 (51.8)	0.975	8 (9.2)	7 (8.4)	0.861
Family H/O HTN: *n* (%) ^¶^	38 (38.8)	28 (33.7)	0.483	26 (29.9)	41 (49.4)	0.009
Family H/O T2D: *n* (%) ^¶^	52 (53.1)	39 (47)	0.415	37 (42.5)	46 (55.4)	0.093
Family H/O CVD: *n* (%) ^¶^	11 (11.2)	16 (19.3)	0.130	4 (4.6)	9 (10.8)	0.126
Central obesity: *n* (%) ^¶^	33 (33.7)	62 (74.7)	0.001	54 (62.1)	79 (95.2)	0.001
Hyperglycemia: *n* (%) ^¶^	19 (19.4)	66 (79.5)	0.001	9 (10.3)	69 (83.1)	0.001
High TG: *n* (%) ^¶^	27 (27.6)	38 (46.3)	0.009	15 (17.4)	44 (53.0)	0.001
Low HDL‐C: *n* (%) ^¶^	30 (30.6)	47 (56.6)	0.001	42 (48.3)	69 (83.1)	0.001
Raised BP: *n* (%) ^¶^	16 (16.3)	33 (39.8)	0.001	13 (14.9)	29 (34.9)	0.003
Anthropometric measures						
Height in cms ^ƥ^	171.0 (166.0–175.0)	165.0 (160.0–169.8)	0.001	160.0 (156.0–164.0)	154.0 (148.0–157.0)	0.001
Weight in kgs ^ƥ^	69.20 (62.0–76.0)	75.0 (70.0–80.0)	0.001	66.0 (56.0–77.0)	72.0 (69.0–79.0)	0.001
BMI kg/M^2 ƥ^	24.05 (21.85–26.27)	28.26 (26.40–30.0)	0.001	25.65 (22.64–28.67)	30.92 (28.69–33.31)	0.001
WC in cms ^ƥ^	88.0 (78.0–95.0)	97.0 (90.0–104.0)	0.001	89.0 (81.0–95.0)	100.0 (91.0–105.0)	0.001
HC in cms ^ƥ^	96.0 (90.75–101.0)	99.0 (95.0–105.0)	0.004	101.0 (93.0–108.0)	105.0 (100.0–109.0)	0.003
Waist‐hip ratio ^§^	0.89 ± 0.065	0.98 ± 0.044	0.001	0.87 ± 0.067	0.94 ± 0.054	0.001
Waist‐height ratio ^§^	0.50 ± 0.058	0.58 ± 0.059	0.001	0.54 ± 0.065	0.64 ± 0.057	0.001
MetS‐related parameters						
FPG (mg/dL) ^ƥ^	86.59 (71.41–97.0)	123.0 (108.0–134.0)	0.001	79.0 (69.0–87.73)	132.0 (113.0–152.0)	0.001
TG mg/dL ^ƥ^	115.96 (82.37–1530)	148.0 (115.0–177.0)	0.001	127.58 (94.0–146.0)	152.0 (114.0–193.0)	0.001
HDL‐C mg/dL ^ƥ^	45.74 (33.33–50.14)	38.0 (33.0–43.0)	0.001	45.0 (39.0–52.0)	38.0 (35.0–43.0)	0.001
Systolic BP (mmHg) ^ƥ^	119.46 (114.0–126.04)	126.0 (117.0–137.0)	0.001	116.0 (107.0–126.0)	128.0 (118.0–133.0)	0.001
Diastolic BP (mmHg) ^§^	72.92 ± 9.40	81.31 ± 8.77	0.001	72.02 ± 8.77	79.73 ± 8.12	0.001

*Note*: Results are expressed as mean ± standard deviation for variables with a normal distribution (^§^), median and interquartile range for variables with a skewed distribution (^ƥ^) based on normality testing by Kolmogorov–Smirnov test, and number with percentage for categorical variables (^¶^). The *p*‐value indicates the differences between non‐MetS and MetS groups according to gender.

Abbreviations: BMI, body mass index; BP, blood pressure; CVD, cardiovascular disease; FPG, fasting plasma glucose; H/O, history of; HC, hip circumference; HDL‐C, high‐density lipoprotein cholesterol; HTN, hypertension; T2D, type 2 diabetes mellitus; TG, triglycerides; WC, waist circumference.

**TABLE 2:** Body composition parameters in non‐MetS and MetS subjects stratified by gender.

Variables	Male (*n* = 181)			Female (*n* = 170)		
	No MetS (*n* = 98)	MetS (*n* = 83)	*p*‐value	No Mets (*n* = 87)	MetS (*n* = 83)	*p*‐value
BIA^**#**^						
BF (%) by BIA^**#** ƥ^	20.79 (16.96–24.03)	25.00 (22.82–26.35)	0.001	33.59 (30.94–35.68)	37.05 (35.85–41.90)	0.001
Fat mass (kg) by BIA^**#** ƥ^	14.86 (10.95–17.25)	19.68 (17.28–22.22)	0.001	22.43 (17.71–26.07)	27.70 (25.52–31.94)	0.001
FMI by BIA^**#** ƥ^	5.28 (3.66–6.26)	7.20 (6.24–8.22)	0.001	8.90 (6.65–10.71)	11.58 (10.43–13.58)	0.001
Lean (%) by BIA^**#** ƥ^	79.20 (75.96–83.03)	74.99 (73.64–77.17)	0.001	65.82 (63.69–69.76)	62.80 (59.17–63.81)	0.001
FFM (kg) by BIA^**#** ƥ^	54.23 (52.34–57.05)	58.23 (56.45–59.86)	0.001	43.02 (41.80–44.79)	46.03 (44.78–47.65)	0.001
FFMI by BIA^**#** ƥ^	18.91 (17.86–20.23)	20.97 (20.16–21.87)	0.001	17.18 (15.82–18.03)	18.94 (18.08–20.08)	0.001
CUN‐BAE						
BF (%) by CUN‐BAE ^ƥ^	21.91 (17.03–24.42)	26.69 (23.86–29.04)	0.001	32.71 (28.79–37.45)	39.71 (36.40–41.84)	0.001
Fat mass (kg) by CUN‐BAE ^ƥ^	14.94 (10.57–19.11)	20.37 (16.62–23.06)	0.001	21.95 (16.96–26.50)	27.72 (24.41–31.48)	0.001
FMI by CUN‐BAE ^ƥ^	4.47 (3.17–5.52)	6.18 (5.19–6.96)	0.001	6.72 (5.35–7.99)	9.00 (7.84–10.55)	0.001
Lean (%) by CUN‐BAE ^ƥ^	78.08 (75.57–82.96)	73.30 (70.95–76.13)	0.001	67.28 (62.54–71.20)	60.28 (58.15–63.59)	0.001
FFM (kg) by CUN‐BAE ^ƥ^	54.04 (50.53–58.24)	55.69 (52.54–59.77)	0.128	42.58 (38.59–49.97)	42.85 (41.87–46.24)	0.151
FFMI by CUN‐BAE ^ƥ^	16.03 (15.10–16.96)	17.01 (16.19–17.87)	0.001	13.18 (12.17–15.44)	14.26 (13.67–15.11)	0.001

*Note*: Results are expressed as a median and interquartile range for variables with a skewed distribution (^ƥ^) based on normality testing by Kolmogorov–Smirnov test. BIA^#^ indicates BF% values by BIA adjusted for age, gender, and BMI using ANCOVA and linear regression. The *p*‐value indicates the differences between non‐MetS and MetS groups according to gender.

Abbreviations: BF, body fat; BIA, bioelectrical impedance analysis; CUN‐BAE, Clínica Universidad de Navarra—Body Adiposity Estimator; FFM, fat‐free mass.

### 3.2 Comparison between CUN‐BAE and BIA for BF

Table 3 compares the BF% values assessed by the BIA and CUN‐BAE methods across all the subjects, non‐MetS subjects, and MetS patients. It includes the means, standard deviations, mean differences, 95% confidence intervals of the differences, and paired sample *t*‐test *p*‐values. The mean BF% values measured by BIA and CUN‐BAE in all the subjects were 28.91 ± 8.02 and 29.22 ± 8.63, respectively. When stratified by gender, the mean BF% values measured by BIA and CUN‐BAE were 22.45 ± 4.48 and 23.21 ± 5.70, respectively, in male subjects and 35.78 ± 4.46 and 35.61 ± 6.33, in female subjects (Table 3). Based on the mean difference values, we did not find significant differences in the means between BIA and CUN‐BAE for BF% in all the subjects or the MetS patients and non‐MetS subjects. However, a significant difference was observed between the means when stratified by gender, especially in total male and MetS male subjects. As shown in Table 3, CUN‐BAE overestimated BF% in total male and MetS male subjects compared with BIA. These results indicate that the CUN‐BAE may be applicable in estimating BF%; however, it performed differently for males and females during gender‐based analyses (Table 3).

**TABLE 3:** Paired *t*‐test to evaluate the mean difference between BIA and CUN‐BAE for BF% measurement in non‐MetS and Mets subjects.

Subjects	BF%_BIA_^#^	BF%_CUN‐BAE_	Mean difference	95% CI of the difference		*p*‐value
				Lower	Upper	
Total Subjects (*n* = 351)	28.91 ± 8.02	29.22 ± 8.63	−0.3103 ± 3.70	−0.6994	0.0787	0.118
Male (*n* = 181)	22.45 ± 4.48	23.21 ± 5.70	−0.7624 ± 2.42	−1.1180	−0.4068	**0.001** *
Female (*n* = 170)	35.78 ± 4.46	35.61 ± 6.33	0.1709 ± 4.66	−0.5349	0.8768	0.633
Non‐MetS Subjects (*n* = 185)	26.35 ± 7.73	26.50 ± 8.34	−0.1500 ± 3.61	−0.6747	0.3745	0.573
Male (*n* = 98)	20.44 ± 4.43	20.70 ± 5.29	−0.2678 ± 1.90	−0.6489	0.1131	0.166
Female (*n* = 87)	33.47 ± 3.24	33.03 ± 6.00	0.4399 ± 4.73	−0.5682	1.4482	0.388
MetS Subjects (*n* = 166)	31.75 ± 7.38	32.24 ± 7.93	−0.4890 ± 3.80	−1.0722	0.0942	0.100
Male (*n* = 83)	24.83 ± 3.70	26.18 ± 4.65	−1.3413 ± 2.68	−1.9267	−0.7558	**0.001** *
Female (*n* = 83)	38.20 ± 4.06	38.31 ± 5.52	−0.1129 ± 4.72	−1.1437	0.9179	0.828

*Note*: All values are mean ± SDs. Values in bold indicate mean difference and paired samples' *t*‐test *p*‐values. BIA^#^ indicates BF% values by BIA adjusted for age, gender, and BMI using ANCOVA and linear regression. Abbreviations as in Table 2.

* Indicates a significant *p*‐value for the difference according to the paired *t*‐test.

### 3.3 Bland–Altman (BA) plot analysis

**FIGURE 1:**
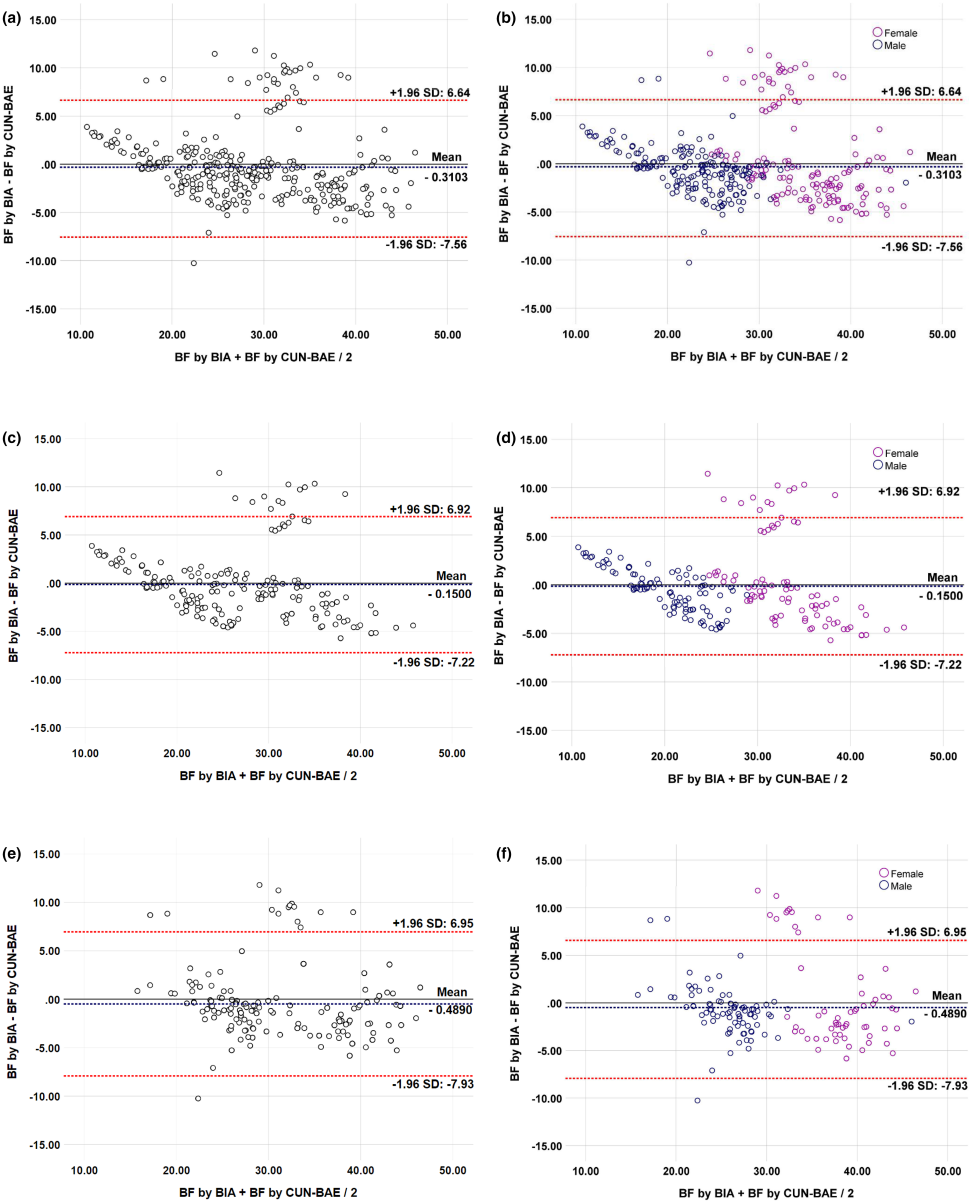
Bland–Altman (BA) plots showing the limits of agreement between BIA and CUN‐BAE methods for BF% in (a) total subjects (*n* = 351). (b) Total subjects (*n* = 351) with male (*n* = 181) and female (*n* = 170). (c) Non‐MetS Subjects (*n* = 185). (d) Non‐MetS Subjects (*n* = 185) with male (*n* = 98) and female (*n* = 87). (e) MetS Subjects (*n* = 166). (f) MetS Subjects (*n* = 166) with male (*n* = 83) and female (*n* = 83). The centre line represents the mean differences between the two methods, and the other lines represent 95% limits of agreement. Analysis of covariance (ANCOVA) and linear regression were used to adjust BF% values by BIA for age, gender, and BMI.

We used BA plots to analyze the absolute agreement between the two methods. These plots show the difference in BF% between the two methods for each sample against the average BF% from the two methods. The vertical axis represents the estimated BF% via CUN‐BAE minus the measured BF% via the BIA method. The horizontal axis represents the mean of the estimated BF% via CUN‐BAE plus the measured BF% via the BIA method divided by two. Three horizontal reference lines were added to the scatterplots to help assess BF% agreement between the BIA and CUN‐BAE methods. The middle line shows the mean difference value, whereas the other lines indicate ± 2 standard deviations from the mean (Table 4; Figure 1). In the BA plot, an equal number of data points above and below the mean difference line indicates good absolute agreement between the two methods. However, more data points above or below the line indicate poor agreement and overestimation or underestimation of BF%. Despite wide limits of agreement, excellent agreement was observed between the two methods for BF% in all the study groups. Further details are available in Table 4 and Figure 1.

**TABLE 4:** Comparison between BIA and CUN‐BAE methods for BF% measurement using Bland–Altman analysis, intra‐class correlation coefficient, concordance correlation coefficient and Kappa coefficient for their absolute agreement, reproducibility, and inter‐rater reliability in the study subjects.

BF% via BIA^#^ versus CUNBAE	Bland and Altman plot analysis		Intra‐class correlation coefficient		Concordance correlation coefficient			Kappa with 95% CI
	Mean bias ± SD of bias	95% limits of agreement	*r* (95% CI)	*p*‐value	*ρ*_c_ (95% CI)	*ρ*	*χ*_a_	
Total Subjects (*n* = 351)	−0.3103 ± 3.70	−7.56 to 6.64	0.948 (0.936–0.958)	0.001	0.900 (0.879–0.918)	0.903	0.996	60.5 (52.0–68.2)
Male (*n* = 181)	−0.7624 ± 2.42	−5.50 to 3.98	0.936 (0.906–0.955)	0.001	0.878 (0.846–0.904)	0.914	0.961	64.3 (52.5–75.2)
Female (*n* = 170)	0.1709 ± 4.66	−8.96 to 9.30	0.780 (0.702–0.837)	0.001	0.637 (0.549–0.711)	0.677	0.941	53.2 (40.1–65.3)
Non‐MetS subjects (*n* = 185)	−0.1500 ± 3.61	−7.22 to 6.92	0.947 (0.929–0.960)	0.001	0.898 (0.867–0.923)	0.901	0.996	46.7 (37.5–63.4)
Male (*n* = 98)	−0.2678 ± 1.90	−3.99 to 3.45	0.960 (0.941–0.973)	0.001	0.869 (0.833–0.898)	0.946	0.919	49.2 (27.8–66.8)
Female (*n* = 87)	0.4399 ± 4.73	−8.83 to 9.71	0.684 (0.517–0.793)	0.001	0.586 (0.445–0.694)	0.631	0.924	36.8 (14.2–59.5)
MetS subjects (*n* = 166)	−0.4890 ± 3.80	−7.93 to 6.95	0.934 (0.910–0.951)	0.001	0.874 (0.834–0.906)	0.879	0.995	51.9 (35.9–65.7)
Male (*n =* 83)	−1.3413 ± 2.68	−6.59 to 3.91	0.864 (0.732–0.924)	0.001	0.783 (0.694–0.849)	0.822	0.952	65.0 (47.3–82.6)
Female (*n* = 83)	−0.1129 ± 4.72	−9.36 to 9.13	0.692 (0.523–0.801)	0.001	0.488 (0.331–0.619)	0.540	0.903	25.5 (5.5–51.1)

Note: BIA^#^ indicates BF% values by BIA adjusted for age, gender, and BMI using ANCOVA and linear regression.

Abbreviations: CI, confidence intervals; *r*, reproducibility; SD, standard deviation; *ρ*, precision; *ρ*_c_, Lin's concordance correlation coefficient; *χ*_a_, accuracy. Other abbreviations are in Table 2.

We used paired *t*‐test *p*‐values to confirm the trend in the BA plots and check for proportional bias and the level of agreement. The paired *t*‐test *p*‐values in Table 3 were statistically insignificant, indicating acceptance of the null hypothesis. This suggests that there is no proportional bias and a good level of agreement between the two measures of BF% (BIA and CUN‐BAE). However, the statistically significant *p*‐value observed in total male and MetS male subjects rejects the null hypothesis, indicating a proportional bias and insufficient agreement between the two measures in a gender‐specific analysis.

### 3.4 Reproducibility and interrater reliability measures

The intraclass correlation coefficient (ICC) results were indicative of good reproducibility between BIA and CUN‐BAE for BF% in total subjects (0.948), non‐MetS subjects (0.947) and MetS subjects (0.934). However, the ICCs between BIA and CUN‐BAE for BF% in female participants of non‐MetS (0.684) and MetS (0.692) groups presented moderate reproducibility (Table 4). Lin's CCC and the 95% confidence interval (95% CI) indicated moderate to good concordance between BIA and CUN‐BAE for BF%. We found moderate concordance between BIA and CUN‐BAE for BF% in all participant groups, except for female participants in the non‐MetS and MetS groups, who showed poor concordance. The degree of interrater reliability measured by the kappa coefficient for the diagnosis of MetS was moderate in the study subjects (total: 60.5%; non‐MetS: 46.7% and MetS: 51.9%). When stratified by gender, the kappa coefficient for the diagnosis of MetS was high in male participants and lowest in female participants with MetS (Table 4).

### 3.5 Bivariate correlation analysis

**FIGURE 2:**
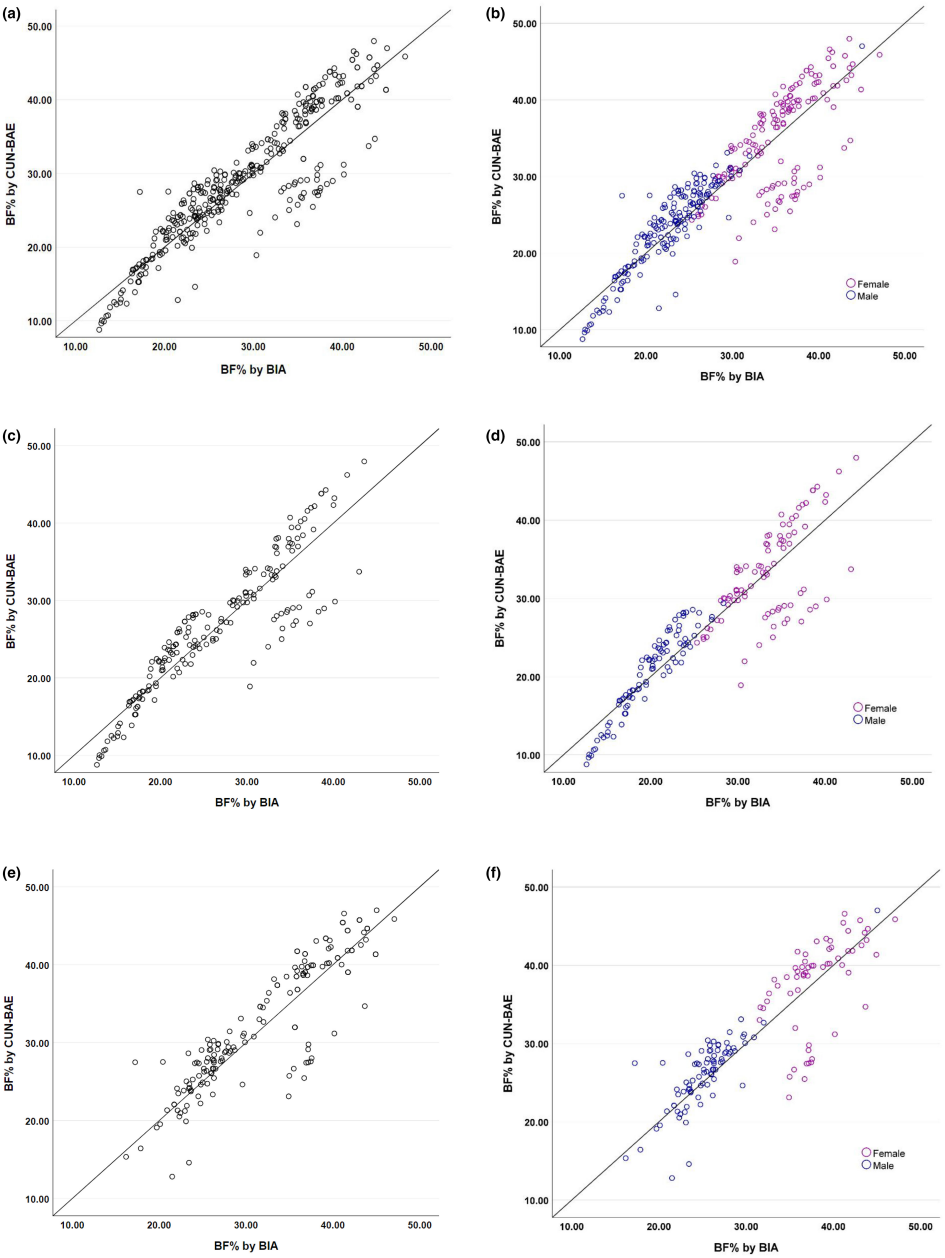
Bivariate correlation analysis between BIA and CUN‐BAE methods in measuring BF%. Correlation between BIA and CUN‐BAE for BF% (a) total study population (*n* = 351, *r* = 0.904, *p* < 0.001). (b) Total study subjects stratified by gender (female: *N* = 170, *r* = 0.688, *p* < 0.001 & male: *N* = 181, *r* = 0.886, *p* < 0.001). (c) Non‐MetS subjects (*n* = 185, *r* = 0.914, *p* < 0.001). (d) Non‐MetS subjects stratified by gender (female: *N* = 87, *r* = 0.568, *p* < 0.001 & male: *N* = 98, *r* = 0.933, *p* < 0.001). (e) MetS subjects (*n* = 166, *r* = 0.878, *p* < 0.001). (f) MetS subjects stratified by gender (female: *N* = 83, *r* = 0.680, *p* < 0.001 & male: *N* = 83, *r* = 0.804, *p* < 0.001). R, Pearson's correlation coefficient; other abbreviations are in Table 2.

We used bivariate correlation analysis to evaluate the relationship between BF% measured via the CUN‐BAE and BIA methods. The correlation coefficients between the BF% measurements via BIA and CUN‐BAE are presented in Figure 2, which shows that the BF% estimated via CUN‐BAE was closely correlated with the corresponding measurements via the standard BIA method. The correlation coefficients (*r*‐values) were 0.904 for all the subjects (female: 0.688; male: 0.886), 0.914 for the non‐MetS subjects (female: 0.568; male: 0.933), and 0.878 for the MetS patients (female: 0.680; male: 0.804), with *p*‐values of <0.001 (Figure 2). Furthermore, we used bivariate correlation analysis to evaluate the degree of association of the BF% estimated by CUN‐BAE with various MetS‐related parameters and compared it with the BF% measured by BIA. In all subjects, the BF% estimated by CUN‐BAE was correlated with most MetS‐related parameters, similar to the BF% measured by BIA in both men and women (Table 5). When the participants were divided into non‐MetS and MetS groups, the BF% estimated by CUN‐BAE showed a stronger association with certain MetS‐related parameters such as WC, systolic blood pressure (SBP), and TG in the male participants of the non‐MetS group. In the MetS group, CUN‐BAE was more strongly correlated with MetS‐related parameters in male subjects than in female subjects. Moreover, BF% estimated by CUN‐BAE was strongly associated with anthropometric variables and marginally with BP, glycemic, and lipid profile parameters in all the subjects (Table 5).

**TABLE 5:** Correlation of BF% measured and estimated by BIA and CUNBAE with MetS‐related parameters.

MetS‐related parameters	Total subjects (*n* = 351)				Non‐MetS subjects (*n* = 185)				MetS subjects (*n* = 166)			
	Female (*n* = 170)		Male (*n* = 181)		Female (*n* = 87)		Male (*n* = 98)		Female (*n* = 83)		Male (*n* = 83)	
	BIA^#^	CUNBAE	BIA^#^	CUNBAE	BIA^#^	CUNBAE	BIA^#^	CUNBAE	BIA^#^	CUNBAE	BIA^#^	CUNBAE
WC	0.693**	0.520**	0.778**	0.772**	0.802**	0.483**	0.870**	0.873**	0.371*	0.322**	0.518**	0.521**
WHR	0.373*	0.220**	0.680**	0.533**	0.411**	0.374**	0.661**	0.539**	−0.129	−0.238*	0.159	0.111
WHtR	0.790**	0.705**	0.860**	0.792**	0.825**	0.601**	0.905**	0.864**	0.566**	0.623**	0.648**	0.593**
SBP	0.311**	0.224**	0.387**	0.351**	0.337**	0.192	0.258**	0.272**	−0.044	−0.018	0.315**	0.235*
DBP	0.403**	0.285**	0.576**	0.493**	0.436**	0.339**	0.392**	0.306**	−0.035	−0.112	0.386**	0.316**
FPG	0.502**	0.427**	0.489**	0.438**	0.165	0.205	0.265**	0.233*	0.108	0.141	−0.142	−0.034
TG	0.300**	0.119	0.361**	0.490**	0.251*	0.182	0.442**	0.515**	0.086	−0.125	0.250*	0.444**
HDL	−0.070	0.002	−0.116	−0.178*	0.109	0.073	−0.035	−0.083	0.024	0.127	0.056	−0.066

*Note*: Data are Spearman's correlation coefficients (rho values) and associated statistical significance (*). BIA^#^ indicates BF% values by BIA adjusted for age, gender, and BMI using ANCOVA and linear regression.

Abbreviations: Abbreviations are as in Tables 1 and 2.

* Correlation coefficient is significant at the 0.05 level (two‐tailed).

** Correlation coefficient is significant at the 0.01 level (two‐tailed).

### 3.6 AUC‐ROC analysis

**FIGURE 3:**
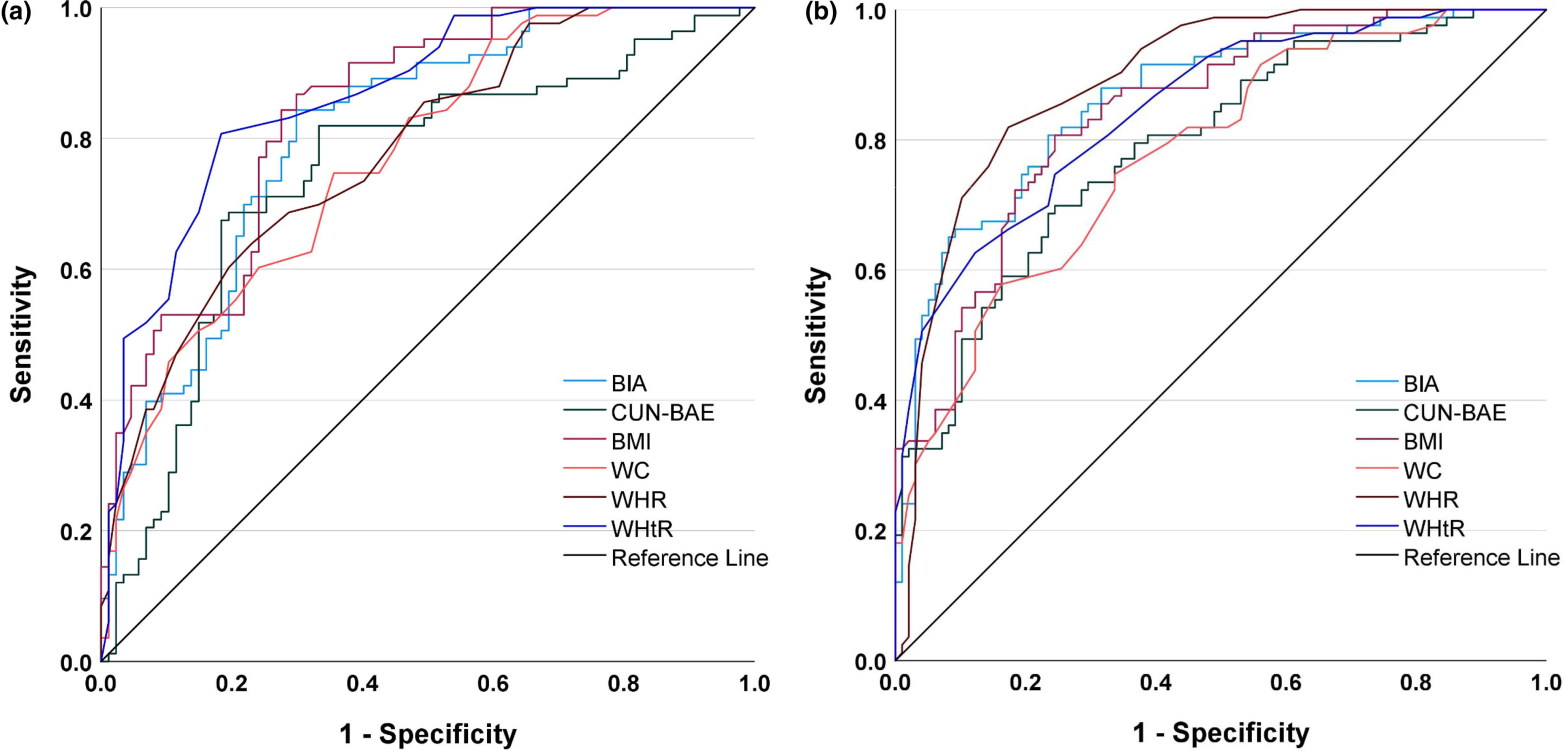
Comparison of the diagnostic accuracies of BIA, CUN‐BAE, and traditional anthropometric measures in identifying MetS in the study population, using AUC‐ROC analysis stratified by gender. (a) Female subjects (b) male subjects. AUCs, ideal cutoffs, sensitivity, and specificity of BIA, CUN‐BAE, and traditional anthropometric measures for identifying MetS are presented in Table 6. AUC, Area under the curve; ROC, Receiver operating characteristics; BIA, Bioelectrical impedance analysis; CUN‐BAE, Clínica Universidad de Navarra‐ Body Adiposity Estimator; WC, Waist circumference; WHR, Waist‐hip ratio; WHtR, Waist‐to‐height ratio.

**FIGURE 4:**
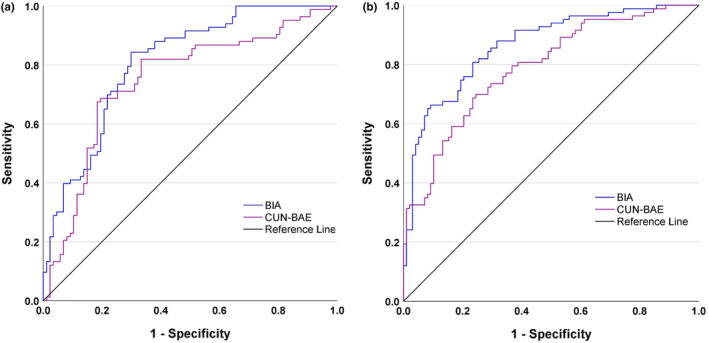
The AUC‐ROC curves comparing the diagnostic accuracy of BIA and CUN‐BAE in identifying MetS based on BF% measurement in the study population, stratified by gender. (a) Female subjects (BIA: AUC: 0.809 & CUN‐BAE: AUC: 0.746) (b) male subjects (BIA: AUC: 0.864 & CUN‐BAE: AUC: 0.790). The ideal cutoffs, sensitivity, and specificity of BIA and CUN‐BAE for identifying MetS are presented in Table 6. AUC, Area under the curve; ROC, Receiver operating characteristics; BF%, Body fat percentage; BIA, Bioelectrical impedance analysis; CUN‐BAE, Clínica Universidad de Navarra‐ Body Adiposity Estimator.

We conducted an AUC‐ROC analysis to evaluate the diagnostic effectiveness of traditional anthropometric measures such as BMI, WC, WHR, and WHtR, as well as BIA and CUN‐BAE. According to the ROC analysis, we found that an AUC of 0.7–1.0 offers the best accuracy in detecting MetS (Table 6). Among the parameters studied, WHR (AUC: 0.891) in men and WHtR (AUC: 0.864) in women showed better diagnostic accuracy than BIA and CUN‐BAE in identifying MetS (Table 6 and Figure 3). Although not superior to these traditional anthropometric measures, BIA and CUN‐BAE also demonstrated slightly better diagnostic accuracy in identifying MetS (with AUCs of 0.809 for BIA and 0.746 for CUN‐BAE in females, and AUCs of 0.864 for BIA and 0.790 for CUN‐BAE in males) (Table 6 and Figure 4). In our gender‐specific analysis, BIA and CUN‐BAE showed stronger predictive ability for MetS in men than in women. Similarly, we found different cutoff values for BIA and CUN‐BAE in men and women for identifying MetS (Figure 4). The optimal cutoff values for identifying MetS with BIA and CUN‐BAE in females and males were found to be >35.4% versus >38.1% and >22.9% versus >24.4%, respectively.

**TABLE 6:** AUCs, ideal cut‐offs, sensitivity, and specificity of BIA, CUN‐BAE, and traditional anthropometric measures for identifying MetS in the ROC analysis.

Variables	Total participants (*n* = 351)	Male participants (*n* = 181)					Female participants (*n* = 170)				
	AUC (95% CI)	AUC (95% CI)	Cutoff	Sen	Spe	YI	AUC (95% CI)	Cutoff	Sen	Spe	YI
BIA^#^	0.696* (0.645–0.744)	0.864* (0.805–0.910)	>22.96	80.7	76.5	0.57	0.809* (0.741–0.865)	>35.34	84.3	70.1	0.54
CUN‐BAE	0.686* (0.635–0.734)	0.790* (0.723–0.847)	>24.36	69.8	75.5	0.45	0.746* (0.674–0.810)	>38.10	68.6	80.4	0.49
BMI	0.831* (0.788–0.869)	0.842* (0.781–0.892)	>26.26	80.7	75.5	0.56	0.839* (0.775–0.891)	>28.08	86.7	70.1	0.56
WC	0.775* (0.728–0.818)	0.779* (0.711–0.837)	>95.00	57.8	83.6	0.41	0.770* (0.700–0.831)	>92.00	74.7	64.3	0.39
WHR	0.831* (0.788–0.869)	0.891* (0.836–0.933)	>0.94	81.9	82.6	0.64	0.780* (0.710–0.839)	>0.92	63.8	77.1	0.40
WHtR	0.842* (0.799–0.878)	0.848* (0.787–0.897)	>0.57	62.6	87.7	0.50	0.864* (0.804–0.912)	>0.59	80.7	81.6	0.62

*Note*: BIA^#^ indicates BF% values by BIA adjusted for age, gender, and BMI using ANCOVA and linear regression.

Abbreviations: AUC, area under the curve; BF%, body fat percentage; BIA, bioelectrical impedance analysis; CI, confidence interval; CUN‐BAE, Clínica Universidad de Navarra‐ Body Adiposity Estimator; ROC, receiver operating characteristics; Sen, sensitivity; Spe, specificity; WC, waist circumference; WHR, waist‐hip ratio; WHtR, waist‐to‐height ratio; YI, Youden index J.

* AUC value is significant at the 0.01 level (two‐tailed).

## 4 DISCUSSION

The term “body composition” applies to the ratio of fat to lean tissue (muscles, bones, body water, and organs) in an individual's body. BF% is a body composition measurement that determines how much of the body's weight is composed of fat. A high BF% is linked to an increased risk of death (Padwal et al., 2016). Thus, estimating BF% can aid in understanding changes in disease conditions concerning body composition. This is relevant from a clinical and public health standpoint because BF% predicts cardiometabolic risk more than BMI (Gómez‐Ambrosi, Silva, Galofré, et al., 2012). Since excess adiposity is an essential determinant for progressing from prediabetes to T2DM to CVD, a reliable estimation of BF% enables early risk stratification (Misra et al., 2010). While many advanced approaches exist to accurately measure the proportion of whole‐body fat, these approaches are not adapted for daily clinical use or large‐scale population studies due to cost, safety, and complexity issues. As a result, several anthropometric‐based equations have been recommended to help estimate the proportion of whole‐body fat. Many reported equations are complicated and require more than 10 anthropometric measurements; some need up to four measurements (Cui et al., 2014). Therefore, a general drawback of the equations is their complexity, which hinders their usefulness in regular use.

Recently, CUN‐BAE has become a newer algorithm widely used for body composition analysis in clinics and research. The CUN‐BAE measures BF% based on an individual's BMI, age, and gender (Gómez‐Ambrosi, Silva, Catalán, et al., 2012). The CUN‐BAE equation may seem more complicated than simple anthropometric measures like BMI, WC, or WHtR, but it is still practical for clinical use. Its added complexity could lead to more accurate and specific results, especially when incorporated into clinical decision‐making systems. Despite its complexity, it can be efficiently implemented in clinical practice by integrating it into Electronic Health Record (EHR) systems or by developing a user‐friendly mobile or web‐based application tailored to healthcare providers. Clinicians could input basic patient data such as age, BMI, and gender into the system, which would then automatically calculate the estimated BF% using the CUN‐BAE equation. These approaches could help in utilizing CUN‐BAE in clinical settings, ranging from primary care to specialized obesity and MetS clinics. Although the CUN‐BAE has the potential to offer a more comprehensive estimate of BF%, there is limited documentation on the use of this technique among Southern Indians who have higher BF% compared to individuals of European descent with similar or lower BMI, due to their tendency to store more fat in their chest and abdomen (Misra et al., 2018).

In a sample of middle‐aged southern Indians, individuals with MetS had higher BF% as assessed by both BIA and CUN‐BAE compared to non‐MetS subjects. Interestingly, women in both the MetS and non‐MetS groups had higher BF% values than men because of their typical fat distribution pattern in the hips and thighs. This “female” fat distribution protects women against metabolic illnesses such as T2DM and atherosclerosis, regardless of their total BF% (Manolopoulos et al., 2010). Furthermore, women can retain more subcutaneous fat than men and are likely to store less visceral fat. Men tend to accumulate less subcutaneous fat, which causes fatty tissue to accumulate more quickly in visceral and ectopic tissues, such as the liver and skeletal muscles. This promotes insulin resistance and disrupts insulin signaling pathways (Sattar, 2013).

Furthermore, we observed that people with MetS have greater fat mass, FMI, and fat‐free mass index (FFMI) values and a lower lean body percentage. The FMI helps provide personalized medical care because some patients with a normal BMI may still have MetS (Bonikowske et al., 2019). Moreover, our research findings support the notion that FFMI could be negatively associated with insulin sensitivity and metabolic health in various populations (Lagacé et al., 2022). Emerging data contradict the widely held belief that having a high FFM protects health. These inconsistencies in conclusions could be due to how the FFMI is represented (relative to weight or squared height [kg/m^2^]). For example, Park et al. reported that FFMI, relative to body weight, reduced the odds of having MetS (Park & Yoon, 2013). In contrast, the FFMI represented relative to the squared height (kg/m^2^), increased the odds of having MetS in a Korean population.

The present study compared the effectiveness of equation‐derived CUN‐BAE in assessing BF% with that of the validated BIA method in the same subjects in both the MetS and non‐MetS groups. Our study findings indicate that using CUN‐BAE to assess BF% produces results comparable to those obtained using the BIA method in gender‐stratified total, non‐MetS and MetS subjects (Table 3). This finding demonstrates that CUN‐BAE is capable of estimating BF% with appropriate precision. Thus, without accurate BF% measurements, CUN‐BAE can be a valuable method for assessing BF%. However, when gender was considered, CUN‐BAE substantially overestimated the BF% in overall male subjects, especially in male MetS subjects, compared with BIA. Another significant finding from this research was that the CUN‐BAE method provided a reliable estimate of BF% compared with BIA, as shown by several measures of agreement, reproducibility and reliability. These included the BA analysis, ICC, CCC, and the kappa coefficient. The results consistently showed good absolute agreement, moderate to good reproducibility, concordance, and inter‐rater reliability across all study subjects. However, the female participants in the MetS and non‐MetS groups exhibited moderate absolute agreement, reproducibility, concordance, and poor inter‐rater reliability. This finding indicates that CUN‐BAE provides the most accurate and precise estimation of BF% compared with BIA in middle‐aged non‐MetS subjects and MetS patients in southern India. However, it performed differently for males and females. While the CUN‐BAE provided reliable estimates for BF in males, its accuracy diminished in females. This difference could be due to variations in fat distribution patterns between genders. Typically, women have a higher percentage of BF and a greater proportion of subcutaneous fat, which may affect the efficacy of the CUN‐BAE. This highlights the necessity of developing customized algorithms to accommodate gender variations resulting from biological variances in future research.

The BF% estimated via CUN‐BAE demonstrated a robust correlation with the BF% measured via BIA (*r* = 0.904) (Figure 2). This strong correlation was also observed in a study by Suliga et al. in the Polish population (*r* = 0.873) (Suliga et al., 2019). The initial development of CUN‐BAE in 2012 by Gomez et al. involved an evaluation of its clinical usefulness in estimating BF% from data on 6510 men and women of European descent aged 18–80 years, which revealed the highest correlation between the BF% estimated by CUN‐BAE and that measured by ADP (*r* = 0.900) (Gómez‐Ambrosi, Silva, Catalán, et al., 2012). Subsequent studies, such as the one by Lara et al. on a group of 40 Caucasian subjects aged 61–84 years and the one by Fuster‐Parra et al. on 3200 Caucasian men and women aged 18–65, consistently reported similar correlations between the BF% estimated by CUN‐BAE and the BF% measured by ADP and BIA (Fuster‐Parra et al., 2015; Lara et al., 2014). This consistent correlation underscores the reliability of CUN‐BAE in estimating BF%. Our study revealed that the BF% estimated via CUN‐BAE was more strongly correlated with MetS‐related parameters than the BF% measured via BIA. This finding highlights the clinical importance of CUN‐BAE in understanding how the estimated BF% might contribute to the changes observed in these MetS‐related parameters concerning body composition. This aspect is particularly significant, as BF% has been shown to correlate more strongly with cardiometabolic risk factors than BMI (Gómez‐Ambrosi, Silva, Galofré, et al., 2012). Moreover, given that actual adiposity is a considerable risk factor for developing prediabetes, MetS, and T2DM (Gómez‐Ambrosi et al., 2011), using CUN‐BAE may be a valuable tool in identifying at‐risk patients.

The AUC‐ROC analysis showed that traditional measures such as BMI, WC, WHR, and WHtR are better at assessing the risk for MetS compared to CUN‐BAE. These measures are easier to estimate and mainly capture central fat. On the other hand, the CUN‐BAE equation provides a more detailed evaluation of total BF%, including both visceral and subcutaneous fat. This difference is important because different types of fat have different implications for metabolic health. While the CUN‐BAE equation may not be superior in predictive power or diagnostic efficacy compared to traditional measures like BMI, WC, and WHtR, it can complement them. The CUN‐BAE equation incorporates multiple variables, and BF% estimated by it may better predict insulin resistance, dyslipidemia, and inflammation compared to traditional anthropometric measures, especially in populations where central obesity is less prominent but overall BF is high. The BF% estimated by CUN‐BAE was similar to that measured by BIA in identifying MetS risk in both women and men. The optimal cutoff points for CUN‐BAE for detecting MetS were 38.1% in female subjects and 24.49% in male subjects. These results were consistent with those reported by Lopez et al. in a Spanish population (28.2% in males and 40.0% in females) (López‐González et al., 2022). However, Peng et al. obtained a closer cutoff point for detecting incident diabetes among the Japanese population (21.96% in males and 30.96% in females) (Peng et al., 2023). The difference in cutoff points may be attributed to differences in body composition, body size, and BF distribution among different ethnic groups.

The most common criteria used in the scientific literature for CUN‐BAE cut‐off points are as follows: normal body fatness is defined as ≤20% of BF in men and ≤30% in women; overweight is defined as 20%–25% of BF in men and 30%–35% in women; and obesity is defined as >25% of BF in men and >35% in women (Gómez‐Ambrosi, Silva, Catalán, et al., 2012; Okorodudu et al., 2010). The Bod‐Pod approach created the CUN‐BAE equation, which predicts metabolic health factors and distinguishes metabolically unhealthy phenotypes (Gómez‐Ambrosi, Silva, Catalán, et al., 2012). Normal‐weight individuals with compromised metabolic health may represent a significant risk group to investigate. In this context, Veronica et al. reported increased BF% values by CUN‐BAE in non‐overweight or non‐obese Spanish subjects (Davila‐Batista et al., 2019). Overall, the absolute agreement, reproducibility, concordance, inter‐rater reliability, and diagnostic efficacy observed between the CUN‐BAE and BIA methods for measuring BF% were good, which agreed with the findings of previous studies, supporting the use of CUN‐BAE as a possible proxy of adiposity among southern Indians.

### 4.1 Strengths and limitations

Our study's main strength lies in the availability of precise measurements of BF% through BIA (QuadScan 4000) in middle‐aged southern Indian subjects. This allowed us to evaluate the performance of CUN‐BAE within non‐MetS and MetS subjects using the same sample. We employed numerous methods to compare the agreement between BIA and CUN‐BAE for measuring BF%. We adjusted the BF% values obtained from BIA for age, gender, and BMI to ensure a more accurate comparison with CUN‐BAE, accounting for the same covariates in both methods. One of the novel findings of our study is the comparison of diagnostic accuracy between BIA and CUN‐BAE methods in identifying MetS based on BF%. We determined the optimal cutoff points for BF% estimated and measured by CUN‐BAE and BIA, respectively, to detect MetS in men and women. This is particularly useful in epidemiological studies where no body composition data are available, and BF% is of interest. However, there are some limitations to our work despite its strengths. The cross‐sectional observation method used may have introduced some bias. Additionally, reference values were measured via BIA, as the gold standard requires more infrastructure. Despite being accurate in most cases, BIA measurements can be influenced by factors such as body position, hydration status, food and drink consumption, ambient air and skin temperature, recent physical activity, and conductance of the examination table, leading to some inaccuracies. Although the sample size was modest, a statistically significant difference was achieved.

Our study shows the usefulness of the CUN‐BAE regression equation for a South Indian population. However, it's important to note that the equation is based on a sample of individuals mainly from Northern European ancestry. This may limit its generalizability to populations with different ethnic, genetic, and lifestyle characteristics. The suitability of regression equations like CUN‐BAE across different populations is a concern in clinical practice and research. Body composition varies significantly between populations due to various factors. An equation derived from a predominantly European sample may not fully capture the unique characteristics of BF distribution in populations such as South Indians. This study evaluated the applicability of the CUN‐BAE equation to a South Indian cohort and found a reasonable level of agreement between estimated and actual BF%. However, future research should prioritize developing and validating body composition regression equations tailored to specific populations. Moreover, the median age of the study population is 44.0–49.0 years, which further limits the generalizability of the findings to other age groups. Future studies should address this by involving a larger, more diverse population to validate CUN‐BAE.

## 5 CONCLUSION

In summary, this study confirms that equation‐derived CUN‐BAE provides similar, accurate, and reliable estimations of BF% in both the MetS and non‐MetS groups compared with the BIA method (QuadScan 4000). Owing to its high accuracy and low‐cost measurement, CUN‐BAE has been developed to estimate BF% for public health purposes. Promisingly, CUN‐BAE agreed well with the BIA method in measuring BF%. It showed good absolute agreement, reproducibility, concordance and inter‐rater reliability in both MetS patients and non‐MetS subjects. However, its usefulness and efficacy are limited when men and women are evaluated separately. This finding needs confirmation in future studies by creating and validating body composition regression equations customized for gender‐specific populations. Based on these findings, we postulate that CUN‐BAE helps measure BF% in routine clinical practice. This equation‐derived measure is easy to apply and can be used as a screening tool, particularly when accessing body composition measurements is difficult.

## AUTHOR CONTRIBUTIONS

All authors conceived and designed the research, performed the experiments, interpreted the results of the experiments, edited, and revised the manuscript, and approved the final version of the manuscript. Conceptualization and design, methodology, investigation, validation, writing—original draft, writing—review & editing, funding acquisition: C.K.E. Formal analysis, validation, drafting the work or revision: C.K.E., G.S.G., D.Y., J.P.S., and B.V.

## CONFLICT OF INTEREST STATEMENT

The authors declare that they have no conflicts of interest.

## ETHICS STATEMENT

This study involved human participants and was approved by the Institute Ethics Committee, Jawaharlal Institute of Postgraduate Medical Education and Research (JIPMER), Puducherry, India (vide letter number JIP/IEC/2018/0301). The participants provided informed consent to participate in the study before taking part.

## Data Availability

The data that support the findings of this study are available from the corresponding author upon reasonable request.
